# Complete Genome Sequence of Sinorhizobium Phage ΦM6, the First Terrestrial Phage of a Marine Phage Group

**DOI:** 10.1128/MRA.01143-18

**Published:** 2018-10-04

**Authors:** Tess E. Brewer, Brian K. Washburn, Jason S. Lynn, Kathryn M. Jones

**Affiliations:** a Department of Biological Science, Biology Unit I, Florida State University, Tallahassee, Florida, USA; b Department of Biological Science Core Facilities, Florida State University, Tallahassee, Florida, USA; Indiana University Bloomington

## Abstract

Sinorhizobium phage ΦM6 infects the nitrogen-fixing rhizobial bacterium Sinorhizobium meliloti. ΦM6 most closely resembles marine phages, such as Puniceispirillum phage HMO-2011, rather than previously sequenced rhizobial phages. The 68,176-bp genome is predicted to encode 121 open reading frames, only 10 of which have similarity to those of otherwise-unrelated Sinorhizobium phages.

## Abstract (precis)

Sinorhizobium phage ΦM6 infects the nitrogen-fixing rhizobial bacterium Sinorhizobium meliloti. ΦM6 most closely resembles marine phages, such as Puniceispirillum phage HMO-2011, rather than previously sequenced rhizobial phages.

## ANNOUNCEMENT

Despite the gains of the genomics era, there are still large gaps in our knowledge of the microbiomes of soils (1). Data are limited on the diversity of bacteriophages that prey upon nitrogen-fixing soil bacteria, which are among the most important microbes in agriculture. Our lack of knowledge of rhizobial phages also limits our understanding of the effects of phage predation on rhizobial survival both in soil and in crop bioinoculants (2–4). ΦM6 is a bacteriophage from a historical collection (2) and infects Sinorhizobium meliloti SU47, a nitrogen-fixing symbiont of Medicago truncatula (barrel medic), and Medicago sativa (alfalfa) (5, 6). Infection of S. meliloti by ΦM6 is dependent on both lipopolysaccharide (7) and the outer membrane protein RopA1 (8). On the S. meliloti SU47-derived laboratory strain S. meliloti 1021 (9), ΦM6 forms very small plaques. It does not fully lyse Sm1021 cultures, reaching titers of only 0.5 × 10^6^ to 3.0 × 10^6^ PFU/ml. ΦM6 DNA was prepared by phenol-chloroform extraction and was sequenced on an Illumina MiSeq platform, as described previously (10). Genome assembly was performed with Lasergene SeqMan Pro version 11.2.1.25 (DNAStar, Madison, WI) from 500,000 MiSeq 2 × 300-bp reads for each of two plaque isolates (plaque isolate sequences were identical), with default read quality control parameters. PhageTerm (11) predicts a 68,176-bp terminally redundant genome with an initiating cleavage predicted at a *pac* site, set as position 1 in the genome. The ΦM6 genome has 42.9% G+C content with no predicted tRNAs (12). ΦM6 has 19 regions of homology with its closest relative, the marine podovirus Puniceispirillum phage HMO-2011 (comprising 4% of the genome [13], aligned with the Mauve plugin [14] in Geneious version 10 [15]). ΦM6 is also similar to several uncultured marine viruses (16). The prediction of 121 open reading frames (ORFs) was performed with GeneMark.hmm (17) and RAST (18). Functions are predicted for 22% of these ORFs (19, 20). The terminase large subunit, portal protein, major capsid protein, and proximal tail proteins are most similar to those of HMO-2011 (13). Because the type of terminase large subunit is often predictive of the type of DNA termini possessed by a phage (21), we determined that the ΦM6 terminase large subunit is a member of terminase superfamily 6 and is 28% identical to that of Escherichia phage HK639. The HK639 genome is circularly permuted with terminal redundancy (21, 22), which is consistent with the ΦM6 termini predicted by PhageTerm. Similar to Pseudomonas phage PA11 (23) and Pseudoalteromonas phage ΦRIO-1 (24), ΦM6 has a genome segment containing 7 ORFs predicted to be involved in the synthesis of peptide bonds (25). This phage module has been proposed to be involved in the modification of host peptidoglycan (24, 25). Although ΦM6 is otherwise unrelated to the rhizobium-infecting myovirus phages ΦM12 (26) and ΦM9 (10), it has 7 ORFs with similarity to those of the ΦM12/N3 genus and 2 with similarity to those of ΦM9. ORF SmphiM6_19 is 32% identical to a predicted tail fiber ORF of ΦM12 (phiM12_124), suggesting that genes encoding host-binding tail fiber proteins may have been shuttled between these phages.

### Data availability.

The genome sequence of Sinorhizobium phage ΦM6 is available in GenBank under accession number MH700630. The fastq files containing the 2 × 300-bp paired-end Illumina MiSeq reads are available from GenBank under Sequence Read Archive (SRA) numbers SRR7788541 for plaque isolate phiM6.1 and SRR7788540 for plaque isolate phiM6.2.
